# Nomenclature and Typification of the Goat Grass *Aegilops tauschii* Coss. (Poaceae: Triticeae): A Key Species for the Secondary Gene Pool of Common Wheat *Triticum aestivum*

**DOI:** 10.3390/plants14152375

**Published:** 2025-08-01

**Authors:** P. Pablo Ferrer-Gallego, Raúl Ferrer-Gallego, Diego Rivera, Concepción Obón, Emilio Laguna, Nikolay P. Goncharov

**Affiliations:** 1 Servicio de Vida Silvestre y Red Natura 2000, Centro para la Investigación y Experimentación Forestal (CIEF), Generalitat Valenciana, Avda. Comarques del País Valencià 114, 46930 Valencia, Spain; flora.cief@gva.es (P.P.F.-G.); emilio.laguna@outlook.es (E.L.); 2 Department of Ecology, Desertification Research Centre, CIDE. Spanish National Research Council (-CSIC), University of Valencia (UV) and Generalitat Valenciana (GVA), 46113 Valencia, Spain; 3 Departamento Biología Vegetal, Facultad Biología, Universidad de Murcia, 30100 Murcia, Spain; drivera@um.es; 4 Instituto de Investigación e Innovación Agroalimentario y Agroambiental (CIAGRO), Escuela Politécnica Superior, Universidad Miguel Hernández, 03312 Alicante, Spain; cobon@umh.es; 5 Institute of Cytology and Genetics, Siberian Branch of Russian Academy of Sciences, Novosibirsk 630090, Russia; gonch@bionet.nsc.ru

**Keywords:** *Aegilops squarrosa*, *Aegilops tauschii*, lectotype, nomenclature, original material, syntype, *Triticum aegilops*, typification

## Abstract

Background: The typification of the name *Aegilops tauschii* Coss. (Poaceae: Triticeae) is revisited. Several authors cited a gathering from Iberia as the locality and Buxbaum as the collector of as the type, but no actual specimens from this collection have been located, nor is there evidence that such a gathering existed. In 1994, van Slageren designated as lectotype an illustration from Buxbaum’s *Plantarum minus cognitarum centuria I* (1728), which, although original material, is not the only element cited in the protologue. The protologue mentions several gatherings, some of which are represented by identifiable herbarium specimens qualifying as syntypes. Methods: This work is based on the analysis of the protologue of *Aegilops tauschii* and the study of specimens conserved in several herbaria. According to the International Code of Nomenclature for algae, fungi, and plants (*ICN*, Shenzhen Code 2018), an illustration does not hold the same nomenclatural weight as preserved specimens cited in the protologue. Therefore, van Slageren’s lectotypification does not comply with Art. 9.12 of the *ICN* and must be superseded. Results: The original material includes multiple elements, and a new lectotype is designated from a specimen at PRC from Azerbaijan.

## 1. Introduction

*Aegilops* L., nom. cons. (Poaceae: Triticeae), is a genus of Eurasian and North American annual grasses [1,2] and forms a significant part of the so-called secondary gene pool of cultivated wheat (*Triticum* L.) [3,4,5,6,7]. According to van Slageren (1994) [8], *Aegilops* comprises 22–23 species [9], and these taxa and their names resulted from taxonomic decisions and from scrutiny of the almost 900 names involved [8]. Traditionally, *Aegilops* species have been classified based on differences in morphology [10,11]. However, opinions about the morphological classification of *Aegilops* species differ among taxonomists [10,11,12], and it is difficult for non-taxonomists to accurately classify *Aegilops* using the established criteria [13].

*Aegilops* is the genus most closely related to wheat (*Triticum*) and provides important genetic resources for its improvement [5,14,15]. According to karyotype analysis, *Aegilops* consists of six genomes: U, C, M, N, S, and D [3,4,16]. Some *Aegilops* species participated in wheat evolution and played a significant role in wheat domestication. A diploid species related to *Aegilops speltoides* Tausch (2*n* = 2*x* = 14, BB genome) was mated with a wild diploid wheat, *Triticum urartu* Thumanjan ex Gandilyan (2*n* = 2*x* = 14, AA genome), to produce tetraploid wheat (*2n* = 4*x* = 28, BBAA genome) [15,17,18,19,20], which was mated again with a diploid *Ae. tauschii* Coss. (2*n* = 2*x* = 14, DD genome) to produce the edible hexaploid bread wheat *Triticum aestivum* L. (2*n* = 6*x* = 42, BBAADD genome) [14,21,22,23,24,25]. This hybridization that gave rise to bread wheat most probably occurred along the southern shores of the Caspian Sea 8000–11,000 years ago [20]. The D subgenome of wheat contains genes responsible for the bread-making quality of wheat flour and related products [26], spring growth habit and earliness [27], and disease resistance [15,28]. The *Ae. speltoides* Tausch most likely is a donor of the B and G genomes of polyploidy wheat species and is related to the Sect. *Sitopsis* (Jaub. et Spach) Zhuk., whereas the D genome is provided by *Ae. tauschii* from Sect. *Vertebrata* Zhuk. emend. Kihara [7,25,29,30,31,32,33].

*Triticum aestivum*, common (bread) wheat, is the most important wheat and one of the most widely cultivated and most successful crop species worldwide. This species has a pivotal role in the global food system. Modern bread wheat shows a remarkably wide geographical distribution and adaptability to various climatic conditions. However, it is treated as a species that has no natural populations and is not found in the wild. In this sense, the existence of archaeological wheat varieties (e.g., *T*. *vulgare* var. *antiquorum*) included currently within *T. aestivum* [34,35,36,37,38,39] confirms that this species always grows in anthropized habitats and is never wild in nature. This species serves as a source of food for 4.5 billion people worldwide, and grain production of about 730 million tons fulfills 20% of the daily protein requirement [40].

The bread wheat D genome progenitor *Ae. tauschii* is a widely distributed [8,41] and genetically diverse species [22,42,43,44]. Based on variation in spike morphology, the species has conventionally been subdivided into two subspecies: *Ae*. *tauschii* subsp. *strangulata* (Eig) Tzvelev and subsp. *tauschii*, of which three varieties have been recognized: var. *tauschii*, var. *anathera* (Eig) K. Hammer (awnless), and var. *meyeri* (Griseb.) Tzvelev (slender, short spikes and develops only 4–8 spikelets per spike) [9,11,45,46]. Members of subsp. *tauschii* develop elongated cylindrical spikelets, while those of subsp. *strangulata* develop moniliform spikes bearing quadrate spikelets [11,45,47,48,49]. However, this classification remains controversial due to the existence of morphologically intermediate types [8,47,50,51], i.e., the same botanical taxa do not always correspond well with genetic relationships [4,22]. The three varieties listed above are currently included in *Ae*. *tauschii* sensu stricto [9]. However, there are several authors who recognize the independence of subsp. *strangulata*. This taxon (subsp. *strangulata*) is distributed from Transcaucasia (Armenia and Azerbaijan) to eastern Caspian Iran [48,52], and it has been widely accepted that it is the donor of the hexaploid wheat D genome [22,42,52,53,54,55,56]. However, the established subspecies structure is not well matched with genetic relationships derived from genotypic characterization. Two distinct phylogenetic lineages, designated L1 and L2, have been recognized [20]. The former coincides with subsp. *tauschii*, but the latter includes, along with subsp. *strangulata*, accessions from some var. *meyeri* formerly assigned to subsp. *tauschii*. The most troublesome taxon is var. *meyeri*, which has been assigned to subsp. *tauschii* on the basis of spike morphology [50] but appears to be genetically more closely related to subsp. *strangulata* [22,42,57,58].

**Figure 1:**
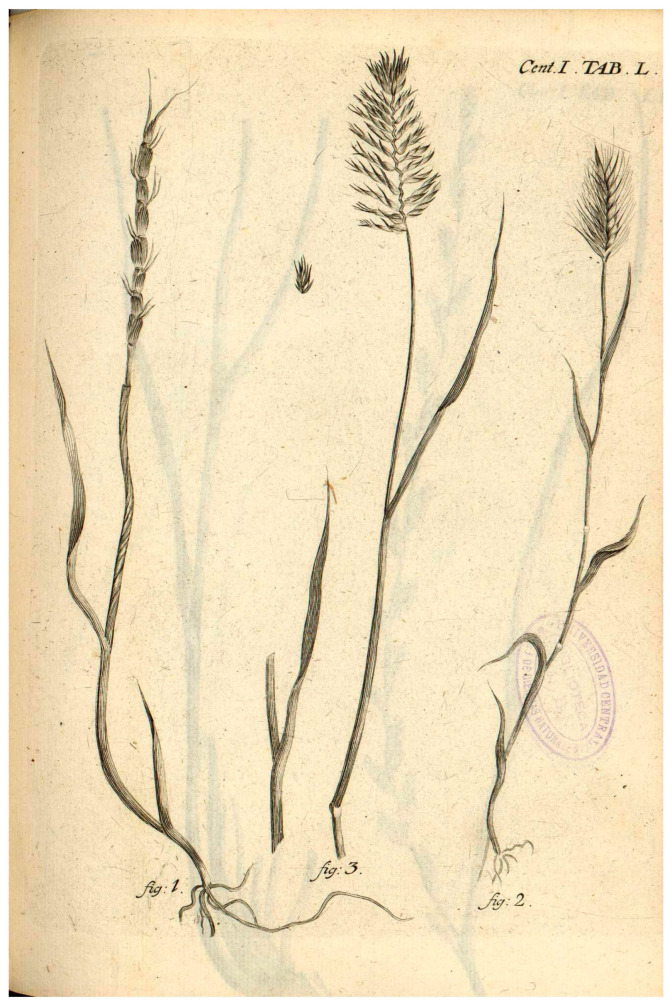
Illustration “Gramen loliaceum spurium, spica crassiore, aristata” from Buxbaum’s *Plantarum minus cognitarum centuria I complectens plantas circa Byzantium & in Oriente observatas* (1728: 31–32, Table 50, Figure 1).

Despite numerous studies on this significant species, a nomenclatural analysis has not yet been conducted. The name *Aegilops tauschii* was lectotypified by Van Slageren (1994) on an illustration “*Gramen loliaceum spurium, spica crassiore, aristata*” from Buxbaum’s (1728) *Plantarum minus cognitarum centuria I, Table L, Figure 1* (Figure 1). However, while this illustration is original material, in the protologue were mentioned several gatherings through the citation of a collector and locality. Some of these gatherings are composed of concrete specimens preserved in herbarium sheets that can unambiguously be recognized as syntypes. Therefore, the illustration does not compete equally with the syntypes that belong to the gatherings that were cited in the protologue, making the previously designated lectotype ineffective according to the International Code of Nomenclature for algae, fungi, and plants (*ICN*, Shenzhen Code of 2018).

In this paper, the original elements used by Cosson in 1849 [59] for the description of *Aegilops tauschii* are discussed. For the sake of nomenclatural stability, a lectotype is proposed to conclusively fix the use of the name. The proposed typification change enables a more precise identification of the element lectotype to fix the name *Aegilops tauschii*, something that was not possible with the illustration previously accepted as the type.

## 2. Results

### 2.1. Nomenclatural Background

The name *Aegilops tauschii* Coss. has been incorrectly treated as a replacement name for *Triticum aegilops* P. Beauv. ex Roem. & Schult. (in Syst. Veg., ed. 15[bis]. 2: 769. 1817) [8,9]. For this reason, the combination “*Triticum tauschii* (Coss.) Schmalh.” (in Fl. Sredn. Južn. Rossii 2: 662. 1897) is considered an illegitimate name (see POWO—Plant of the World Online) [9]. *Aegilops tauschii* should be treated as the name of a new taxon because there is no reference in the protologue to a basionym or replaced synonym and because this was not the author’s presumed intent (see *ICN* Art. 41.4) (see Cosson, 1849) [59]. *Triticum aegilops* P. Beauv. ex Roem. & Schult. is simply a superfluous and illegitimate replacement name for *Aegilops squarrosa* L. (see Linnaeus, 1753: 1051). Roemer & Schultes (1817: 769) cite “*Aegilops squarrosa* Willd. Spec. IV. p. 944.” and Willdenow (1806: 944), apart from citing the second edition of Linnaeus’s *Species Plantarum*, as “A. [Aegilops] spica subulata aristis longiore. *Sp*. *pl*. 1489.” (i.e., Linnaeus, 1763: 1489), which does not differ noticeably from the first, and also seems just to be adopting Linnaeus’s 1753 name. However, Cosson (1849: 69) did not mention Roemer & Schultes or Willdenow, but only cited “Æ. squarrosa, Schreb. Gram. fasc. II. 44. t. 27. f. 2—Tausch, in Flora (1837) 108, non auct.”; moreover, by the “non auct.” Cosson clearly excluded Linnaeus’s name and also Roemer & Schultes’s and Willdenow’s usage of it.

In conclusion, *Aegilops tauschii* was validly published as the name of a new species that Schreber (1719: 44) and Tausch (1837: 108) misidentified as *Aegilops squarrosa* L. Roemer & Schultes (1817), and Willdenow (1806), may also have misidentified *Ae*. *squarrosa* L. This is the situation addressed in *ICN* Art. 41 Note 3.

### 2.2. Typification of the Name

In the protologue, Cosson mentioned: “ÆGILOPS TAUSCHII (NOB.)—*Æ. squarrosa*, Schreb. Gram. fasc. II. 44. t. 27. f. 2.—Tausch, in Flora (1837) 108, non auct.—Boiss. voy. Esp. 683.—*Æ. cylindrica var. taurica*, Roem. et Schult. syst. II. 771.—*Æ. caudata*, Sm. et Sibth. fl. Græc. I. p. 76. 76 ex adnot. non L.—*Gramen loliaceum spurium spica crassiore aristata*, Buxbaum. I. 31. cent. I. t. 50.”, followed by a description in Latin: “Spica cylindrica, e spiculis 6–8 composita. Glumis etiam spiculæ terminalis muticis, ovato-subquadratis, haud ventricosis, apice truncatis edentulis, glumellis brevioribus. Glumellis inferioribus spicularum lateralium aristatis, vel quibusdam muticis bidentalis truncatisve, in spicula terminali aristis longioribus. (1). Maio-junio.” [trans.: cylindrical spike, composed of 6–8 spikelets. The glumes of the terminal floret are also unarmed, ovate-subquadrate, and not bloated, with truncate, toothless apices, and the lower glumellae are shorter. The lower glumellae of the lateral florets are awned, or some are unarmed, bidentate, or truncated, while in the terminal floret, the awns are longer. (1). May–June.].

In the protologue was also included the provenance and some gatherings, as “Habitat in Iberia (Buxbaum. loc. cit.; Wilhelms in herb. Gay). In Tauria (Tausch, loc. cit.). In graminosis et aridis prope *Elisabethpol* Georgiæ Caucasicæ (Hohenacker, un. it. 1834. primum sub nomine Æ. cylindrica, Host dein in schedula emendata Æ. squarrosa.)—In horto Parisiensi culta.” [trans.: Habitat in Iberia (Buxbaum, *loc. cit*.; Wilhelms in Gay’s herbarium). In Tauria (Tausch, *loc. cit*.). In grassy and arid places near Elisabethpol in the Caucasian region of Georgia (Hohenacker, collected in 1834, originally under the name *Æ. cylindrica* Host, later corrected on the label to *Æ. squarrosa*).—Also cultivated in the Paris garden.] Finally, a brief diagnosis in French was also included: “Cette plante se distingue des diverses formes de l’*Æ*. *squarrosa* par les glumes non renflées-ventrues, tronquées même celles de l’épillet terminal” [trans.: This plant is distinguished from the various forms of *Æ. squarrosa* by the glumes that are not swollen-ventricose, even those of the terminal floret, which are truncated].

Are the gatherings mentioned by Cosson in the protologue truly relevant: i.e., (1) in Iberia, Buxbaum; (2) in Iberia, Wilhelms; (3) in Tauria, Tausch; and (4) in graminosis et aridis prope Elisabethpol Georgiae Caucasicae, 1834, Hohenacker? In addition, according to the protologue, this plant is also cultivated in the botanical garden in Paris (“In horto Parisiensi culta”). Unfortunately, Cosson did not select any element as the typus.

On the other hand, without designating it as the type, the first part of the provenances and gatherings mentioned by Cosson in the protologue, “In Iberia”, with or without the name “Buxbaum”, has been cited as a “type” of *Ae. tauschii* by some authors (see e.g., Bor, 1970; Cope, 1982). However, in all cases the typification is ineffective (see below). Later, Davis (1985: 238) [60], however, cited all elements from Cosson (see above) as the syntypes, thus indicating that he did not regard the notation from Bor (1970) [61], Tzvelev (1976) [49], and Cope (1982) [62] as a lectotypification of the name.

Concretely, Bor (1970: 195) only mentioned “Typus: Habitat in Iberia, Buxbaum”. Later, Cope (1982: 595) indicated an alleged type as “type” “Type: “Iberia”, *Buxbaum*”. These two references included a locality, “Iberia”, and a collector, “Buxbaum”. Thus, Bor’s indication (1970: 195) could certainly satisfies Art. 7.10 and 7.11 of the *ICN* [63] and constitutes an effective lectotype designation, because he clearly indicates the “type element” mentioned in Art. 7.11 (“… if the type element is clearly indicated…”) since an element can be considered as “…a single specimen or gathering…or illustration…” as indicated in the Art. 40.3 of the *ICN*. Moreover, the lectotypification proposed by Bor (1970) [61] could also be further narrowed to a single specimen by a “second-step” lectotypification according to Art. 9.17 of the *ICN*. However, despite intensive searches by the authors over the years, no material from this gathering (“Buxbaum—In Iberia”) could be traced. In conclusion, this type of selection is more likely a result of a mechanical process, where the first element cited in the protologue is chosen, rather than a deliberate selection of the most appropriate element to serve as the nomenclatural type. This could undoubtedly lead to future confusion if a clear typification is not provided.

In the protologue, this locality, “Iberia”, and collector, “Buxbaum”, were indeed cited as follows: “Habitat in Iberia (Buxbaum, loc. cit.)”. In Buxbaum’s work (1728: 31–32) [64], under the polynomial “Gramen loliaceum spurium, spica crassiore, aristata”, the following provenance is indicated: “In montibus apricis Iberiæ Iulio” [In sunny places in the mountains of Iberia, flowering in July]. However, he was referring to the Caucasus region, not the Spanish-Portuguese location [The Centuria deals with plants found “circa Byzantium & in Oriente observatas” and was published in St. Petersburg.] Nevertheless, no gathering was mentioned. The mention of this “gathering” (locality and author) does not appear to be associated with any herbarium material. If such herbarium material existed, it would be the lectotype of the name, and if there were multiple specimens from the “gathering” indicated in the protologue, namely, originating from “Iberia” and collected by “Buxbaum”, one of the specimens could be selected as the second-step lectotype (as mentioned above).

Johann Christian Buxbaum (no later than 5 October 1693–7 July 1730) was a German physician, botanist, entomologist, and traveler. In 1721, Peter the Great, tsar of Russia, invited him to accept a position as botanist in the Apothecary Garden at the Medical Collegium in St. Petersburg. In 1725 he became a full member of the St. Petersburg Academy of Sciences and Arts and a professor at the Academic Gymnasium. In his capacity as a physician, Buxbaum in 1724 was called upon to accompany Alexander I. Rumyantsev to Constantinople in a Russian diplomatic mission to the Ottoman Empire (Turkey). He used this opportunity to visit Greece. On his way back from Constantinople, he visited Asia Minor; travelling through Baku and Derbent, he reached Astrakhan to return, finally, to St. Petersburg (in 1727). Buxbaum published his work *Plantarum minus cognitarum centuria I complectens plantas circa Byzantium & in Oriente observatas* in 1728, and it is likely that he studied *Ae. tauschii* during his trip in 1724, from which he created his iconography (Table 50, Figure 1).

The herbarium of J.C. Buxbaum is absent from St. Petersburg, Russia. Invited by Peter the Great to oversee the Apothecary’s Garden of the Medical Collegium on Voroniy (Aptekarsky) Island, now known as the Komarov Botanical Institute in St. Petersburg, Buxbaum cataloged the flora of St. Petersburg and its surrounding areas. However, his collections are not stored there. After the end of the contract with the St. Petersburg Academy of Sciences, Buxbaum took his herbarium collection to “the Electorate of Saxony (Germany)”.

The Herbarium of St. Petersburg University (LECB) is one of the oldest and largest university herbaria in Russia, containing an extensive collection of herbarium sheets, including many from historically significant collections. However, the herbarium was organized after Buxbaum’s departure from St. Petersburg.

However, it appears that such a gathering does not exist, or at least we have not been able to locate it in any of the herbaria consulted. In this regard, the type indication provided by Davis (1985: 238) [60] seems to support this, as he refers to all the syntypes in a general manner: “Syntypes: [USSR] in Iberia (Buxbaum, Wilhelms). In Tauria (Crimea), Tausch. In graminosis et aridis prope Elisabethpol, Georgiae Caucasicae, 1834, Hohenacker.—In horto Parisiensi culta (all P)”, but without selecting any of them as a lectotype. This suggests that he did not consider the “lectotypification” by the previously cited authors as valid [61,62]. It seems that the reference to “Iberia” and “Buxbaum” in the protologue reflects what Buxbaum indicated geographically rather than a specific collection, or at least a collection that was not seen by Cosson, who might have only intended to record the locality mentioned by Buxbaum. It is important that the protologue also mentions “In horto Parisiensi culta”.

At this point, it is necessary to consider the interpretation of the type by van Slageren (1994) [8]. Van Slageren (1994: 13, 326–329) designated the lectotype of *Ae. tauschii* Coss., based (erroneously) on the “basionym” *Triticum aegilops* P. Beauv. ex Roem. & Schult., as: “Lectotype (nov.): the illustration of Table 50, Figure 1 in Buxbaum’s (1728) *Plantarum minus cognitarum Centuria 1*.”. The illustration “Gramen loliaceum spurium, spica crassiore, aristata” from Buxbaum’s (1728: 31–32, Table 50, Figure 1) (see Figure 1) shows a complete plant, including leaves and inflorescences. This illustration certainly represents the traditional and current concept of the name *Ae. tauschii*.

Buxbaum cites the phrase name from Scheuchzer’s *Agrostographia* (1719: 42), “Gramina spicata, spica simplici, loliacea, spuria,” but the accompanying illustration in Scheuchzer’s work clearly refers to tussock grasses or bunch grasses (*Parapholis incurva* (L.) C.E.Hubb.). Von Trinius (1822: 229) [65] wrongly identified Buxbaum’s description and plate (i.e., Table 50, Figure 1) as *Ae. cylindrica*, as “1132. Gr. [Gramen] loliaceum spurium, spica crassiore, aristata. *Buxb*. *Cent*. 1. *p*. 31. Table 50. *f*. 1. Aegilops cylindrica”.

However, this lectotypification is ineffective according to Art. 9.12 of the *ICN*. For the purposes of lectotypification, Art. 9.12 makes clear that syntypes must be selected over original material that was not cited in the protologue and cited and uncited illustrations. As such, the lectotypification by van Slageren (1994) [8] must be revised, and the name *Ae. tauschii* retypified with a specimen belonging to one of the collections cited in the protologue.

In summary, according to the protologue, there are four potential gatherings whose specimens (if they exist) can be considered as syntypes: (1) “Iberia and Buxbaum”; (2) “Iberia and Wilhelms in herb. Gay)”; (3) “In Tauria (Tausch, loc. cit.)”; and (4) “In graminosis et aridis prope *Elisabethpol* Georgiæ Caucasicæ (Hohenacker, un. it. 1834. primum sub nomine Æ. cylindrica, Host dein in schedula emendata Æ. squarrosa.)”.

We have found several relevant herbarium sheets at some herbaria (see below). The specimens that are part of the gathering (exsiccatum) mentioned in the protologue as “In graminosis et aridis prope *Elisabethpol* Georgiæ Caucasicæ (Hohenacker, un. it. 1834. primum sub nomine Æ. cylindrica, Host. dein in schedula emendata Æ. squarrosa.)” are accompanied by the same printed label, annotated as “Aegilops cylindrica. Host./squarrosa Lin. (manuscript)/Unio itiner. 1834./T. Fr. Hohenacker./In graminosis et aridis prope Elisabethpol Georgiae caucasicae./May.” The locality “Elisabethpol” was the name of a city located in the historical region of Transcaucasia, now known as Ganja, in Azerbaijan. Rudolph Friedrich Hohenacker (1798–1874) edited many series of exsiccates between 1841 and 1874, containing plants collected by himself from Caucasia and adjoining areas and collected by others from Abyssinia, Chile, India, Italy, Mesopotamia, Surinam, Sweden, and Ukraine.

Belonging to this exsiccatum, we have found specimens at BM, E, G, JE, K, L, NY, OXF, P, PRC, TUB, US, and W; with barcodes or 2D codes such as the following: BM000086310, BM000086320, E00363053, E00363065, G00191957, G00191953, G00191954, G00191954, JE00036237, K000743772, L1204591, L1204594, L1204595, P02637004, P02637006, P02637013, PRC 454714, W 1889-0230741, W 1916-0013528, W 1889-0017016, W 1912-0020328, W 1889-0230741A, W 0000471, and W 1889-0251338. This is consistent with what was published by van Slageren (1994: 340), who indicates that this gathering “near Elisabethpol (=Kirovabad), *Hohenacker s.n*.” is preserved in the following herbariums: “BM, E, G, G-BOIS, JE, K, L, NY, OXF, P, P-CO, PRC, TUB, US, W”. Additionally, van Slageren (1994) did not cite material from any of the other collections mentioned in the protologue (i.e., “Iberia and Buxbaum”, “Iberia and Wilhelms in herb. Gay” or “In Tauria (Tausch, loc. cit.)”).

On the other hand, the sheet with barcode BM000086326 contains two plants of this species and a handwritten label annotated as “Triticum, Linn./(Aegilops, L./squarrosa, L.)/O. N. Agrosticaceae./Iberia caucasica./(Hohenacker.)”. This specimen could be a duplicate from the exsiccatum, but confirming this is not possible.

**Figure 2:**
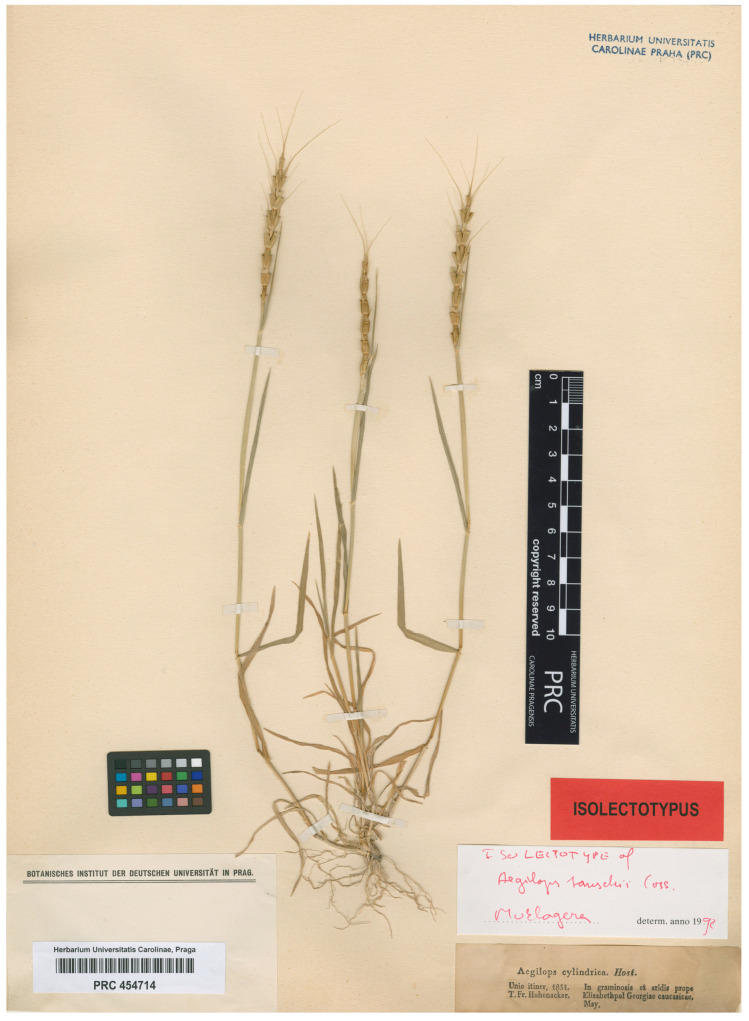
Lectotype of *Aegilops tauschii* Coss. (PRC barcode PRC 454714). Image courtesy of the herbarium PRC, reproduced with permission.

In conclusion, among the original material, we select the specimen at PRC as the lectotype of the name *Aegilops tauschii* (Figure 2). This specimen matches the traditional concept and current use of the name and shows diagnostic features of *Ae*. *tauschii* (e.g., culms up to 40 cm tall; leaf sheath glabrous but with a ciliate margin; ligule up tp 1 mm; leaf blade 4–6 × ca. 0.3 cm, scabrous, adaxial surface pilose; spike cylindrical, up to 8(–10) cm long, only slightly tapering towards the apex, with 7–10 spikelets; rachis sinuate; spikelets cylindrical, ca. 9 mm, with three or four florets; glumes 4–6 mm, scabrid, leathery, 7–9-veined, apex of glumes of lateral spikelet truncate or slightly toothed with a thickened rim and an adaxial mucro, apex of glumes of apical spikelet obtuse with a central mucro; lemma lanceolate, 5-veined; first lemma ca. 7 mm; triangular awn up to 4 cm; apex of lemmas of lateral spikelets thickened and with a mucro (in basal part of the spike) that may develop into an awn of up to 4 cm long on the adaxial side of the lemma apex, often with a small tooth on the abaxial side, apex of lemmas of the apical spikelet with a slender, up to 5.5 cm long) [8,49,60,61,62,66].

*Aegilops tauschii* Coss., Notes pl. Crit.: 69. 1849 ≡ *Triticum tauschii* (Coss.) Schmalh., Fl. Sredn. Južn. Rossii 2: 662. 1897 ≡ *Patropyrum tauschii* (Coss.) Á. Löwe, Biol. Zentralbl. 101: 206. 1982—Lectotype (designated here): Azerbaijan, “In graminosis et aridis prope Elisabethpol Georgiae caucasicae” [Ganja], May 1834, *R*. [*T*. on the herbarium sheet] *Fr. Hohenacker s.n*. (Unio itiner. 1834), PRC barcode PRC 454714. Isolectotypes: BM000086310, BM000086320, E00363053, E00363065, G00191957, G00191953, G00191954, G00191954, JE00036237, K000743772, L1204591, L1204594, L1204595, P02637004, P02637006, P02637013, NY 05154305, OXF, US03445480, W 1889-0230741, W 1916-0013528, W 1889-0017016, W 1912-0020328, W 1889-0230741A, W 0000471, W 1889-0251338. For an image of the lectotype, see Figure 2.

## 3. Materials and Methods

This work is based on the analysis of the protologue of *Aegilops tauschii* and the study of specimens conserved in several herbaria. All original elements (specimens and the illustration previously designated as the “lectotype”) used to describe this species have been carefully evaluated to determine the precise taxonomic application of the name. The identity of the designated type is verified against the traditional and current use of the name. Acronyms of the herbaria consulted (i.e., BM, E, G, JE, K, L, LE, LECB, MW, NY, OXF, P, PRC, TUB, US, and W) are according to Thiers (2025) [continuously updated] [67]. All *ICN* Articles cited in the text refer to the *Shenzhen Code* [63].

## Data Availability

Data are contained within the article.
